# Role of intraflagellar transport protein IFT140 in the formation and function of motile cilia in mammals

**DOI:** 10.1007/s00018-025-05710-z

**Published:** 2025-05-10

**Authors:** Yi Tian Yap, Jiehong Pan, Jian Xu, Shuo Yuan, Changmin Niu, Cheng Zheng, Wei Li, Ting Zhou, Tao Li, Yong Zhang, Michael J. Holtzman, Gregory J. Pazour, Rex A. Hess, Christopher V. Kelly, Aminata Touré, Steven L. Brody, Zhibing Zhang

**Affiliations:** 1 Department of Physiology, Wayne State University, 275 E Hancock Street, Detroit, MI 48201 USA; 2 Division of Pulmonary and Critical Care Medicine, Department of Medicine, Washington University School of Medicine, St. Louis, MO USA; 3 Department of Occupational and Environmental Health, School of Public Health, Wuhan University of Science and Technology, Wuhan, Hubei China; 4 School of Nursing, School of Public Health, Yangzhou University, Yangzhou, Jiangsu China; 5 Institute of Reproductive Health, Tongji Medical College, Huazhong University of Science and Technology, Wuhan, 430030 China; 6 Program in Molecular Medicine, University of Massachusetts Chan Medical School, Worcester, MA USA; 7 Department of Comparative Biosciences, College of Veterinary Medicine, University of Illinois, 2001S. Lincoln, Urbana, IL USA; 8 Department of Physics and Astronomy, Wayne State University, Detroit, MI USA; 9 Université Grenoble Alpes, INSERM U1209, CNRS UMR 5309, Institut pour l’Avancée des Biosciences (IAB), Team Physiology and Pathophysiology of Sperm cells, 38000 Grenoble, France; 10 Department of Obstetrics and Gynecology, Wayne State University, Detroit, MI USA

**Keywords:** Cilia, Fertility, Intraflagellar transport, Sperm

## Abstract

Cilia are microtubular structures extending from the surface of most mammalian cells. They can be categorized as motile cilia and primary sensory cilia. Both types possess intraflagellar transport (IFT) machinery, composed of unique protein complexes that travel along the microtubules to deliver proteins for ciliary and flagellar assembly, disassembly, and homeostasis. Although the role of IFT in primary cilia formation has been well studied, little is known about its role in mammalian motile cilia assembly. We generated conditional knockout mice by breeding floxed *Ift140* mice with the *FOXJ1-*Cre transgenic mouse line to specifically delete *Ift140* from cells that assemble motile cilia. Mice with *Ift140* deficiency did not have laterality defects or gross; however most died prior to sexual maturity. Those mutants that survived to adulthood were completely infertile. Males demonstrated abnormal spermatogenesis associated with reduced sperm count and motility, together with short length flagella, and abnormal morphology. Cilia length was diminished in the epithelial cells of the efferent ductules and airways. Cilia from cultured tracheal epithelial cells were also short and had reduced beat frequency (CBF). Ultrastructural studies revealed the presence of inner and outer dynein arms, but an abnormal central apparatus, and the accumulation of particles within the cilia. Overall, the short length and abnormal localization of ciliary proteins in *Ift140* conditional mutants resulted in inadequate cilia function despite proper localization of the dynein motor complexes. We propose a key role of *Ift140* for motile cilia assembly in certain tissues and suggest that genetic alterations of *IFT140* could be associated with motile ciliopathies.

**Supplementary Information:**

The online version contains supplementary material available at 10.1007/s00018-025-05710-z.

## Introduction

Cilia are microtubular structures present at the surface of most mammalian cells [1]. The two major subtypes of cilia are primary cilia and motile cilia which differ both structurally and functionally. Primary cilia are cellular protrusions commonly found on the apical region of many cell types and predominantly serve sensory functions. They are comprised of 9 microtubule doublets, giving them the characteristic “9 + 0” structure. On the other hand, motile cilia are present in sperm and the multiciliated cells lining the brain ventricles, the respiratory tract, the oviduct in females, and the efferent ducts in males. The typical structure of a motile cilium is comprised of 9 microtubule doublets and a pair of central microtubules, resulting in the “9 + 2” configuration. Essential characteristic structures present in the motile cilia are dynein arms that facilitate ciliary beat and movement [2].

Even though it was reported that ribosomes are also present in the motile cilia [3], it has been well established that the components of the cilia are produced within the cell body and then transported to the developing cilium. Intraflagellar transport (IFT) is the cargo transport system that facilitates the assembly and maintenance of both primary and motile cilia. The bidirectional IFT transport was discovered by Rosenbaum and colleagues using differential interference contrast microscopy to observe trafficking in paralyzed *Chlamydomonas* mutants [4]. Further studies showed that IFT is required for the assembly of all types of cilia, including mammalian [5, 6]. Two multimeric IFT complexes, traveling in opposite directions, enable bidirectional particle transport along the axoneme. IFT complex A consists of IFT43, IFT121, IFT122, IFT139, IFT140, and IFT144 for retrograde transport. While IFT complex B comprises of 16 proteins subcategorized into IFT-B1 (IFT22, IFT25, IFT27, IFT46, IFT52, IFT56, IFT70, IFT74, IFT81, IFT88) and IFT-B2 (IFT20, IFT38, IFT54, IFT57, IFT80, and IFT172) for anterograde transport [7]. During anterograde transport, cargo proteins are conveyed from the cytoplasm to the tips of the cilia and sustain cilia growth and homeostasis. Subsequently, turnover products are transported from the tip back to the base of the cilia for recycling through retrograde transport [8, 9]. Dedicated motor proteins, kinesins and dyneins, mediate anterograde and retrograde IFT trafficking, respectively [10, 11]. IFT has been predominantly studied in primary cilia with recent focus on genetic variants. Defects in the IFT machinery are associated with many human syndromic ciliopathies, including Bardet–Biedl syndrome, Sensenbrenner syndrome, Mainzer–Saldino syndrome, and Jeune syndrome [12–16], as well as isolated retinal degeneration, polycystic kidney disease, and male infertility [17–20]. Primary ciliary dyskinesia (PCD) is the genetic disorder caused by motile cilia dysfunction, resulting in a range of clinical manifestations affecting the upper and lower respiratory tract, reproductive system, and left–right body asymmetry [21]. However, the precise role of IFT genes in motile cilia assembly and maintenance and related diseases such as PCD, including male infertility remains unclear. With the exception of *Ift74* [22], most patients with pathogenic *IFT* variants are not known to have respiratory defects. Studies in mice show that mutations in *Ift* genes like *Ift88*  [23] significantly impact respiratory and other multiciliated cell types, suggesting similar phenotypes should be observed in the human population. However, perinatal lethality and other severe non-respiratory pathologies resulting from the same mutations in *IFT* genes could obscure respiratory dysfunction. In addition, it is possible that pathogenic variants in humans are hypomorphic and may not be strong enough to result in significant respiratory complications. Thus, the importance of *Ift* in multiciliated cells is relatively unexplored.

We have previously studied several mouse models with conditional deficiency in selective *Ift* genes induced in adult mice during spermiogenesis, with a focus on the IFT-B complex components. We reported that disruption of *Ift20, Ift25*, *and Ift27* in male germ cells resulted in infertility [24–26]. Likewise, IFT81 male mice were infertile associated with reduced IFT-B components in testes, and others, including IFT20, IFT25, IFT27, IFT57, IFT74, and IFT88, but there was no change in the IFT-A complex protein IFT140 [27]. We reported that conditional deletion of IFT140 in spermatocytes and spermatids resulted in infertility [28], implicating a requirement for IFT-A component in mammalian motile ciliogenesis in sperm. The requirement of specific IFT components in other motile cilia has not been widely studied. To address this, we deleted a floxed allele of *Ift140* in cells with motile cilia using *FOXJ1-Cre*.

Forkhead box J1 (Foxj1) serves as the master regulator of motile ciliogenesis. This transcription factor is almost exclusively expressed in ciliated cells present in the respiratory tract, choroid plexus, ependyma, oviduct, efferent ducts, and embryonic nodal regions [29–32]. Inhibition of Foxj1 results in compromised assembly of motile cilia in zebrafish and Xenopus [33]. In mice during lung development, the expression of *Foxj1* was first detected at embryonic day 14.5, just prior to the appearance of cilia [29]. Mice mutants lacking Foxj1 expression demonstrate the absence of cilia in airways, left–right axis defects, hydrocephalus, and infertility [30, 34]. A similar phenotype including left–right defects, hydrocephalus, and perinatal mortality is observed in mice deficient in genes regulated by Foxj1 that are commonly deficient in PCD, such as the murine orthologues of DNAH5, DNAI1, DNAAF5, CCDC39, and CCDC40 [35–40]. As such, the established mouse line containing the *FOXJ1-Cre* transgene provides a tool for investigating the role of genes in the mammalian motile cilia [41].

To expand our knowledge regarding the role of IFT in motile cilia, we bred floxed *Ift140* mice with *FOXJ1-Cre* mice. This breeding strategy yielded mice lacking the specific IFT140 protein exclusively in motile cilia. *Ift140 *^*flox/flox*^*; FOXJ1*-Cre (*Ift140* cKO) mice survived to adulthood, but were infertile, with the males exhibiting defects in spermatogenesis and altered motile cilia in the efferent ductules. The remaining *Ift140* cKO mice displayed delayed growth, and death at less than three weeks of age. Cilia from tracheal epithelial cells (mTEC) cultured from the *Ift140* cKO mice were less numerous and exhibited decreased ciliary beat frequency (CBF). Electron-dense particles were observed in the cilia of *Ift140* cKO mTEC. Sperm associated gene 16L (SPAG16) and sperm associated gene 17 (SPAG17), key central pair components, were mislocalized in the epithelial cells. These findings strongly support the involvement of IFT140 in the formation of mammalian motile cilia.

## Results

### Generation of a *FOXJ1*-Cre mediated *Ift* mutant mouse model

To investigate the role of IFT140 in motile cilia, we bred floxed *Ift140* with *FOXJ1-Cre* mice to generate conditional knockout mice, where the *Ift140* gene was specifically disrupted in the motile cells, all in a C57BL/6 background (Supplemental Fig. 1). Genotyping confirmed the acquisition of homozygous mutant mice (Supplemental Table 1). *Ift140*^*flox/*+^; littermates that were *FOXJ1-Cre*^±^ or *Ift140*^*flox/flox*^ mice negative for *FOXJ1-Cre* were used as controls.

**Fig. 1:**
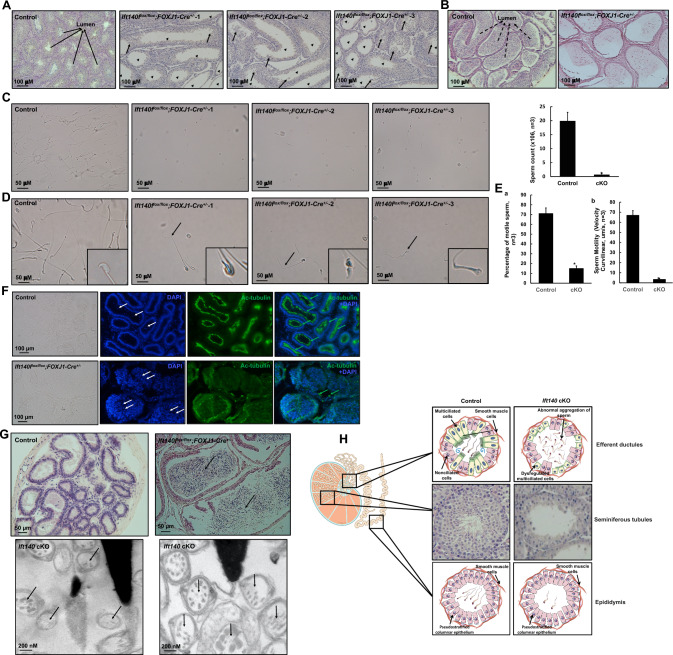
Sperm formation was affected in the surviving conditional *Ift140* cKO mice. **A** Representative testicular histology from a control mouse and three *Ift140* cKO mice. Notice that the lumen of the seminiferous tubules in *Ift140* cKO mice were highly dilated (arrow heads); a number of degenerated germ cells were sloughed in the lumen (arrows); **B** Representative epididymis histology from a control mouse and a *Ift140* cKO mouse. Notice that the lumen was filled with sperm in the control mouse (dashed arrows) while few sperm cells were detected in *Ift140* cKO mice. **C** Significantly reduced sperm count in *Ift140* cKO mice. Left: representative images at low magnification of epididymal sperm collected from a control mouse and three *Ift140* cKO mice. Notice that the sperm density was lower in the three cKO mice when the samples were prepared in the same dilution; right: statistical analysis of sperm count from three control mice and three *Ift140* cKO mice. *p < 0.01. **D** Abnormal sperm morphology in *Ift140* cKO mice. **a** Representative high magnification sperm images from a control mouse and three *Ift140* cKO mice. Most sperm from *Ift140* cKO mice had bent tails (arrows); **b** Average length of sperm flagella of the control and *Ift140* cKO mice. 50 sperm with straight tails were randomly selected from four control and three *Ift140* cKO mice and the tail length were measured. *P < 0.01. **E** Significantly reduced sperm motility in the *Ift140* cKO. **a** Percentage of motile sperm;** b** Sperm motility. *P < 0.01. **F** Reduced cilia signal on the efferent ductules of the *Ift140* cKO mice compared to controls. Representative images of immunofluorescence staining conducted on efferent ductules closer to the testes side collected from a control mouse and a *Ift140* cKO mouse, using an anti-acetylated tubulin antibody. Strong cilia signal (green arrows) was visible in the control mouse. However, few cilia signals were detected in *Ift140* cKO mice. The lumen was dilated and more sperm (white arrows) were present in the efferent ductules of *Ift140* cKO mouse. **G** Examination of the efferent ductules of the *ift140* cKO mice. Upper panel: Normal efferent ductules histology was observed in the control mice. However, the lumen was highly dilated in *Ift140* cKO mice and more sperm accumulated here (arrows)*.* Lower panel: Representative TEM images of sperm from efferent ductules of a *Ift140* cKO mouse. The arrows point to sperm axonemes with disrupted structure. **H** A cartoon to summarize the reproductive phenotypes observed in the absence of IFT140 motile cilia. Dysfunction of motile cilia in the efferent ductules in the *Ift140* cKO mice likely caused a failure of fluid absorption, which results in lumen dilation in both proximal efferent ductules and the seminiferous tubules. The accumulation of fluid inside the seminiferous tubules disrupted spermatogenesis. The formed sperm cells also accumulated at the proximal efferent ductules. Figure is adapted from images on opensource database (https://www.imaios.com/en/e-anatomy/anatomical-structures/body-of-epididymis-1541221040#) and SMART Servier medical art (https://smart.servier.com/)

**Table 1:**
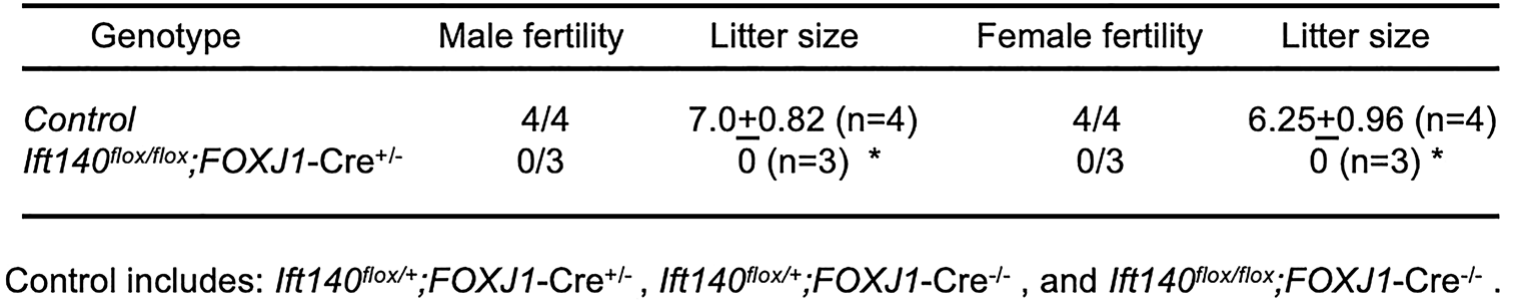
Fertility and fecundity of the control and *Ift140*^*flox/flox*^*;FOXJ1*-Cre mutant mice

To test the fertility, four-month-old mice with the indicated genotypes were bred to four-month-old wild-type animals for two months. Litter sizes were recorded for each mating

^*^P < 0.01

### *Ift140 *cKO mice that survived to adulthood exhibited infertility had spermiogenesis defects

The first generation of *Ift140* cKO mice (*Ift140*^*flox/flox*^*; FOXJ1-Cre*^±^) did not exhibit gross abnormalities such as hydrocephalus, and they had normal left–right asymmetry. However, none of the homozygous males and females *Ift140* cKO mice were able to sire pups over a period of more than two months of breeding (Table 1). Spermatogenesis was evaluated in the *Ift140* cKO adult males. Histological examination of the testes revealed an abnormal spermatogenesis process in the *Ift140* cKO mice. In control mice, immature germ cells were aligned along the basal region of the tubules, while mature sperm were found within the tubule lumen (Fig. 1A). Conversely, in the *Ift140* cKO mice, the seminiferous tubules displayed an enlarged lumen and were lined with a thinner layer of cells (Fig. 1A). Numerous mature sperm were concentrated in the cauda epididymis of control mice (Fig. 1B). However, in the *Ift140* cKO mice, the cauda epididymal lumen contained fewer sperm, along with sloughed round spermatids and cell debris (Fig. 1B). Consistently, sperm count was significantly reduced in the *Ift140* cKO mice (Fig. 1C). Further examination of sperm morphology at high magnification using light microscopy revealed that sperm from the control mice exhibited normal morphology whereas most sperm from the *Ift140* cKO mice were short and had bent tails with altered head morphology (Fig. 1D). Additionally, while sperm from the control mice demonstrated normal motility (Supplemental movie 1, Fig. 1E), the few sperm from the *Ift140* cKO mice exhibited reduced motility (Supplemental movie 2, Fig. 1E).

In wild-type mice, motile cilia are present in the efferent ductules and the beating of these cilia is known to be responsible for stirring or creating luminal turbulence for proper reabsorption of nearly 90% of the fluid [42]. It has been reported that dysfunction or loss of motile cilia in the efferent ductules disturbs the luminal homeostasis, causing sperm accumulation, occlusion of the efferent ducts, and buildup of fluid within the testis, all of which result in male infertility [43–45]. Consequently, the efferent ductules were examined. A strong and continuous ciliary signal was observed in the epithelial cells along the lumen in control mice (Fig. 1F**,** upper panel). However, in the *Ift140* cKO mice, the ciliary signal was scattered along the lumen (Fig. 1F**,** lower panel**)**. Histology of the efferent ductules was also examined. In contrast to the control mice, the *Ift140* cKO mice exhibited a much larger lumen with sperm trapped inside (Fig. 1G, upper panel). The few TEM cross-sectional images of the axoneme of an *Ift140* cKO mouse suggested a disrupted central pair (Fig. 1G, lower panel). The major phenotype observed in male *Ift140* cKO mice was summarized in Fig. 1H.

### *Ift140 *cKO mice exhibited growth retardation and early death after the first generation

**Fig. 2:**
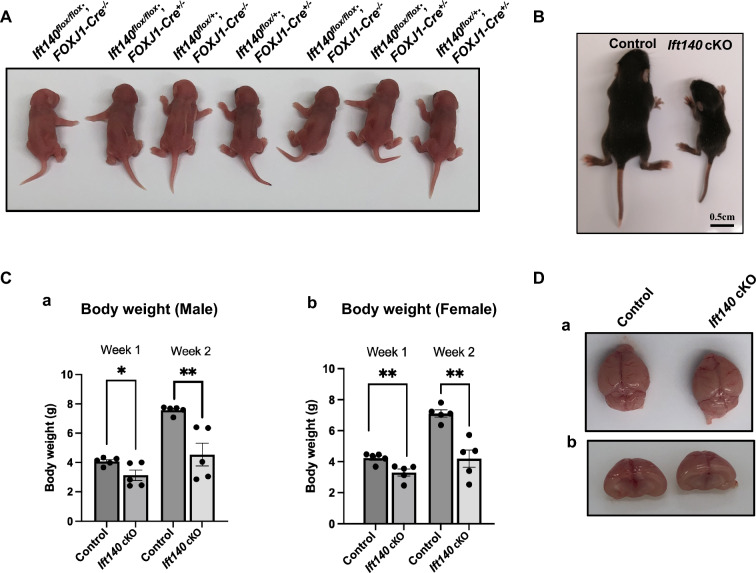
*Ift140 *cKO mice exhibited growth retardation and early death. **A** No difference was observed in one-day-old mice from all genotypes. A representative image of a one-day-old litter and the pups showing the same size in all genotypes. **B** A representative image of a two-week-old control mouse and *Ift140* cKO littermate. Notice that the size of the cKO mouse was much smaller than the control. **C** Body weight of one-week-old and two-week-old control and *Ift140* cKO mice. For both male (**a**) and female (**b**), the body weights were significantly lower in the cKO mice; the difference was greater at two-week-old. All cKO mice died before three weeks old after the second generation. **D** Morphology of the brains isolated from control and *Ift140* cKO mice. No obvious hydrocephalus was found in the *Ift140* cKO mice

Although the first generation of *Ift140* cKO mice did not exhibit gross abnormalities, starting from the second generation, all the homozygous mutant mice began to show growth retardation and eventually died before reaching three weeks of age. To closely monitor this process, the body weight of *Ift140* cKO mice was measured weekly. Upon birth, no differences were observed among the various genotypes (Fig. 2A). However, by approximately one week of age, the *Ift140* cKO mice exhibited smaller size, and by two weeks of age, their size was dramatically less than their control littermates (Fig. 2B). Both male and female (Fig. 2Cb) *Ift140* cKO mice had significantly lower body weights compared to controls at both weeks 1 and 2 of age (Fig. 2Ca, Cb**)**. All *Ift140* cKO mice succumbed before reaching three weeks of age. No instances of hydrocephalus (Fig. 2D) or situs inversus were observed in these mice and cilia in the brain ventricles appeared unaffected as indicated by immunostaining of ependymal cilia in control and *Ift140* cKO mice (Supplement Fig. 2). Histology of major organs, including the kidney and liver, was examined in the control and *Ift140* cKO mice. No cystic changes found in mice with primary cilia defects were observed in any of these organs (Supplemental Fig. 3).

### *Ift140* cKO mice had abnormal cilia formation in the airway epithelial cells

**Fig. 3:**
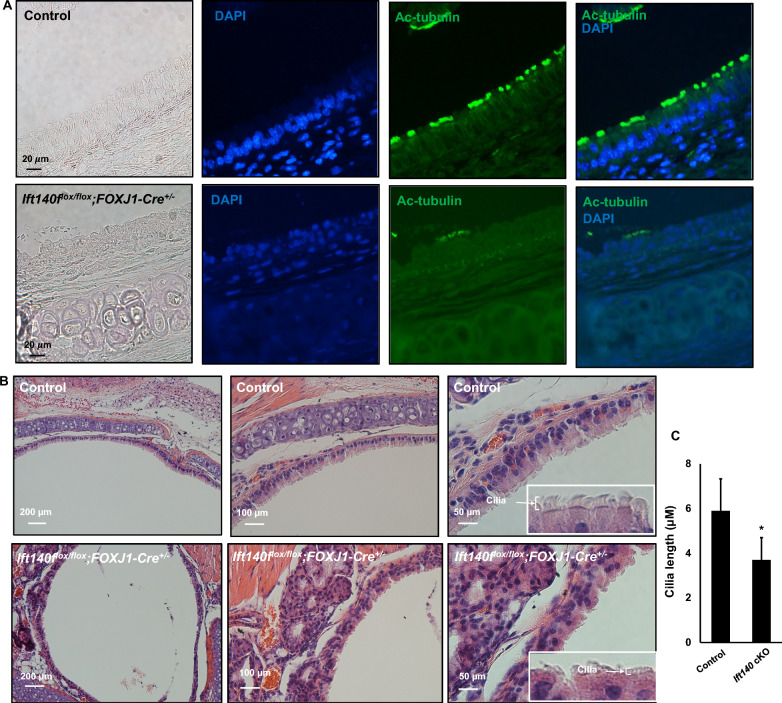
Abnormal cilia formation in tracheal epithelial cells from *Ift140 *cKO mice. **A** Immunofluorescence staining of trachea shows abnormal cilia in the *Ift140* cKO mice. The cilia signal is visualized by staining with anti-α-tubulin antibody. The cilia signal in the cKO mouse was dramatically reduced. **B** Histological examination of the trachea from control (upper) and *Ift140* cKO mice (lower). Fewer cilia were present in the cKO mice and the cilia appeared to be shorter (inserts). **C** Average length of the cilia is indicated in B. Three control and three cKO mice were analyzed. For each mouse, cilia were randomly selected from three regions, and the lengths were measured. * P < 0.05

**Fig. 4:**
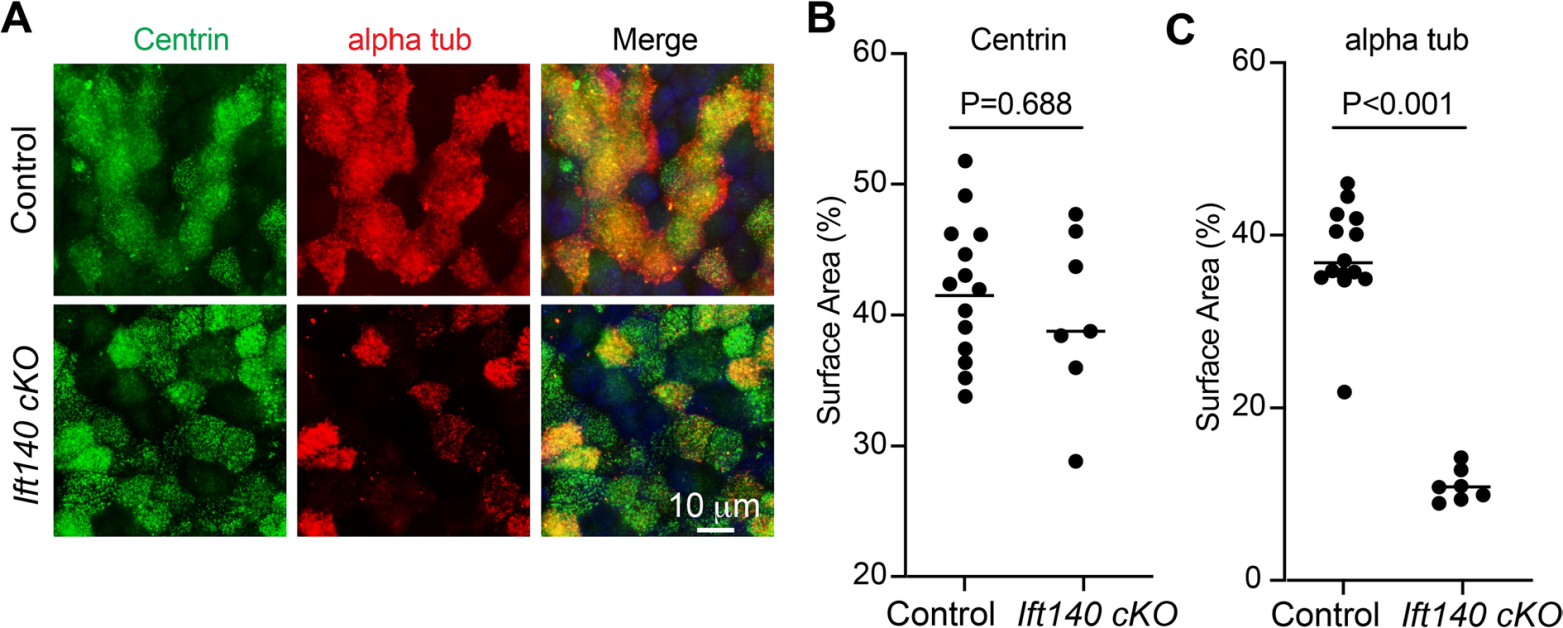
Ciliogenesis in cultured tracheal epithelial cells from *Ift140 *cKO mice is delayed. Tracheal epithelial cells were cultured and co-stained with anti-centrin antibodies to label basal bodies and anti-α-tubulin to label cilia. No difference was found in the number of centrin positive cells, however the α-tubulin signal was dramatically reduced in *Ift140* cKO mice. Left: representative images showing centrin and α-tubulin signals in the control and *Ift140* cKO mice; Right: Statistic analysis of the centrin and α-tubulin positive area

**Fig. 5:**
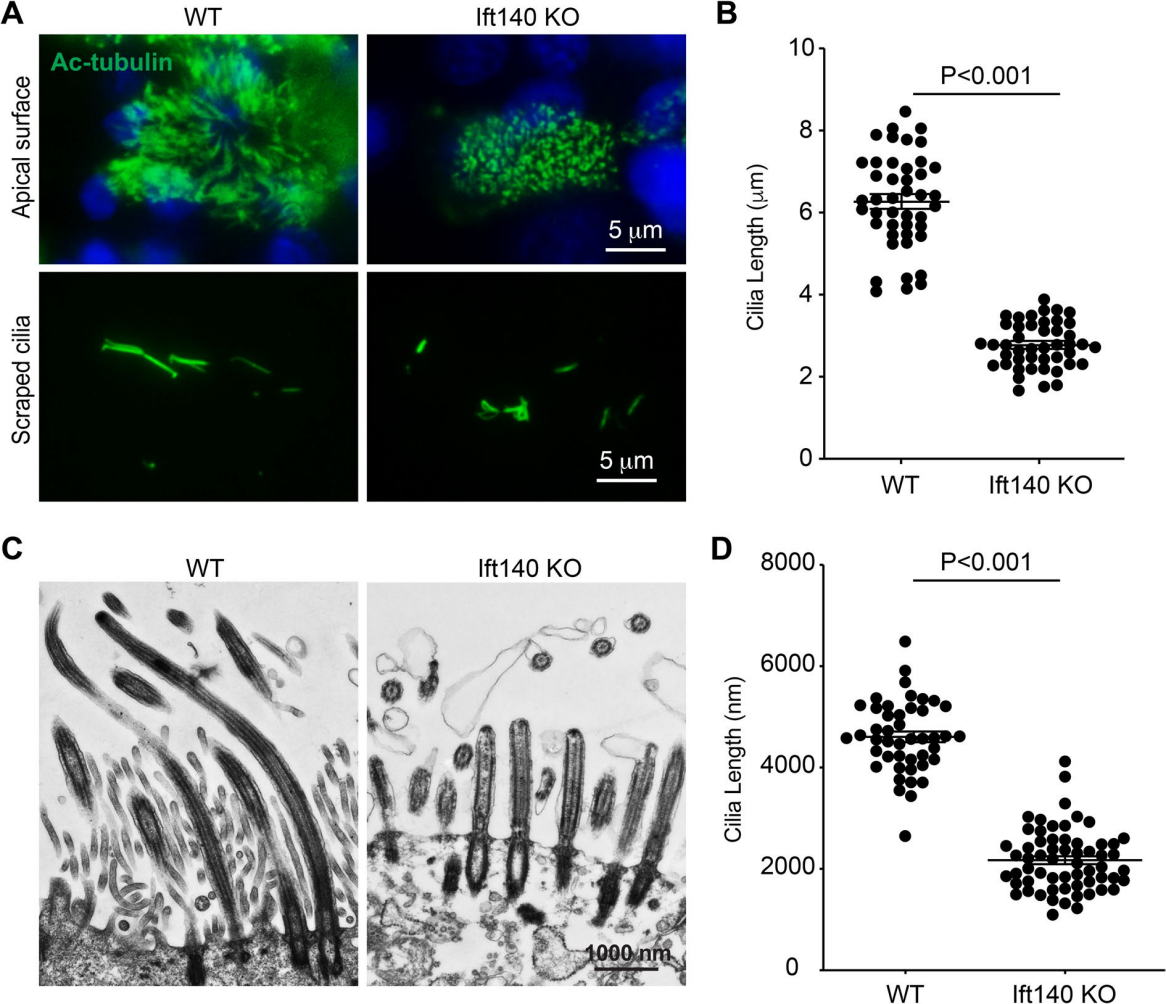
Reduced cilia length in tracheal epithelial cells from the *Ift140* cKO mice. **A** Examination of cilia length by immunofluorescence staining. Left: representative images of the staining results; right: Statistic analysis of cilia length measurements from the images. **B** Examination of cilia length by TEM. Left: representative TEM images; right: Statistic analysis of cilia length as evaluated from the TEM images

Despite the survival of the first generation of *Ift140* cKO mice to adulthood, all mutants were infertile, suggesting ineffective function of motile cilia in the efferent ductules and oviduct. The remaining homozygous mutant mice died before reaching three weeks of age, posing a challenge for analyzing all organs with cilia of these small mice. Therefore, we analyzed only the trachea and lungs. Immunofluorescence staining revealed a near continuous acetylated tubulin signal in the epithelial cells lining the trachea, bronchi, and intrapulmonary airways of control mice (Fig. 3A, upper panel). Conversely, the *Ift140* cKO mice exhibited only scattered ciliary signals (Fig. 3A, lower panel). Histological examination of the trachea unveiled morphological differences between control and *Ift140* cKO mice. In control mice, the lumen was lined with cilia projecting into the lumen (Fig. 3B, upper panel). However, in *Ift140* cKO mice, fewer cilia were observed in the epithelial cells, and these cilia were shorter (Fig. 3B, lower panel; Fig. 3C). To further assess the cilia observed in the trachea of mice, mTEC were cultured from both control and *Ift140* cKO mice. Analysis of ciliogenesis of cultured cells from control and cKO mice showed similar centriologenesis and basal body docking, but decreased cilia number in the cKO preparations (Fig. 4). Abundant multiciliated cells were observed in control mTEC (Supplemental Fig. 4, left three panels). In contrast, *Ift140* cKO mTEC had fewer multiciliated cells, often with sparse cilia (Supplemental Fig. 4, right two panels). Examination of cilia length assessed by immunofluorescence staining and TEM in mTEC cultures revealed that *Ift140* cKO had noticeably shorter cilia compared to control mTEC (Fig. 5).

**Fig. 6:**
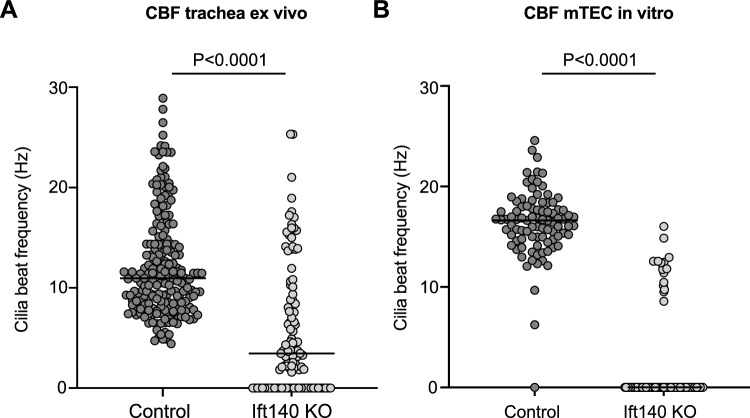
Reduced cilia beat frequency in tracheal epithelial cells of the *Ift140 *cKO mice. **A** Ciliary beat was analyzed about 20 h after trachea isolation and maintenance of the samples in DMEM. **B** Ciliary beat was analyzed in cultured mTEC cells in two independent preparations of *Ift140* cKO and control cells. In both preparations, ciliary beat was reduced in the *Ift140* cKO mice. N = 4

Cilia from the *Ift140* cKO mTEC were also functionally abnormal, as evidenced by the reduced cilia beat frequency (CBF) (Fig. 6). Cilia beat frequency was analyzed in cells isolated by scraping cells from tracheal surface and in cultured mTEC. Compared to controls, ciliary beating frequency was significantly reduced in cells isolated from the *Ift140* cKO trachea (Fig. 6A, Supplemental Fig. 5, Supplemental movie 3) and in cultured mTEC (Fig. 6B, Supplemental movie 4).

### *Ift140 *cKO mouse tracheal epithelial cells exhibit abnormally accumulated particles within cilia

**Fig. 7:**
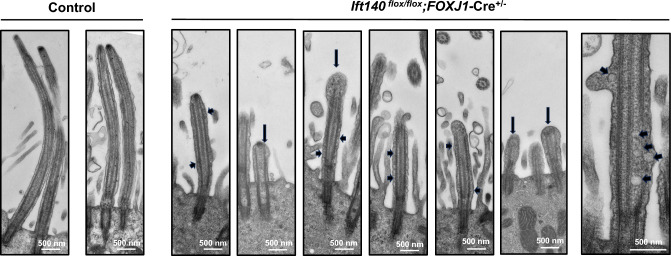
Abnormal protein trafficking in the cultured tracheal epithelial cells (mTEC)* from Ift140 *cKO. TEM was conducted in cultured mTEC from the control and *Ift140* cKO mice. The mTEC cells from the control mice showed long and smooth cilia (left). However, in the cells from the *Ift140* cKO mice, the cilia were not only shorter, they trapped particles and bulges (arrowheads). In some cilia, the tips were swollen (arrows), a typical feature of defective IFT

Mutations in *Ift* genes are known to cause changes in cilia ultrastructure due to altered trafficking of ciliary proteins. Electron micrographs of control mTEC revealed typical ciliary structures (Fig. 7). In contrast, *Ift140* cKO mTEC exhibited the presence of electron-dense particles that appeared to accumulate within the cilium, as well as swollen tips in some cilia (Fig. 7, Supplemental Fig. 6) which is consistent with observations of IFT-B mutations in the motile cilia of *Chlamydomonas*, *Tetrahymena*, and the sensory cilia of *C. elegans* [46].

### Inactivation of *Ift140* in the motile cilia results in normal assembly of axonemal dynein motors, but impaired delivery of central pair apparatus proteins

**Fig. 8:**
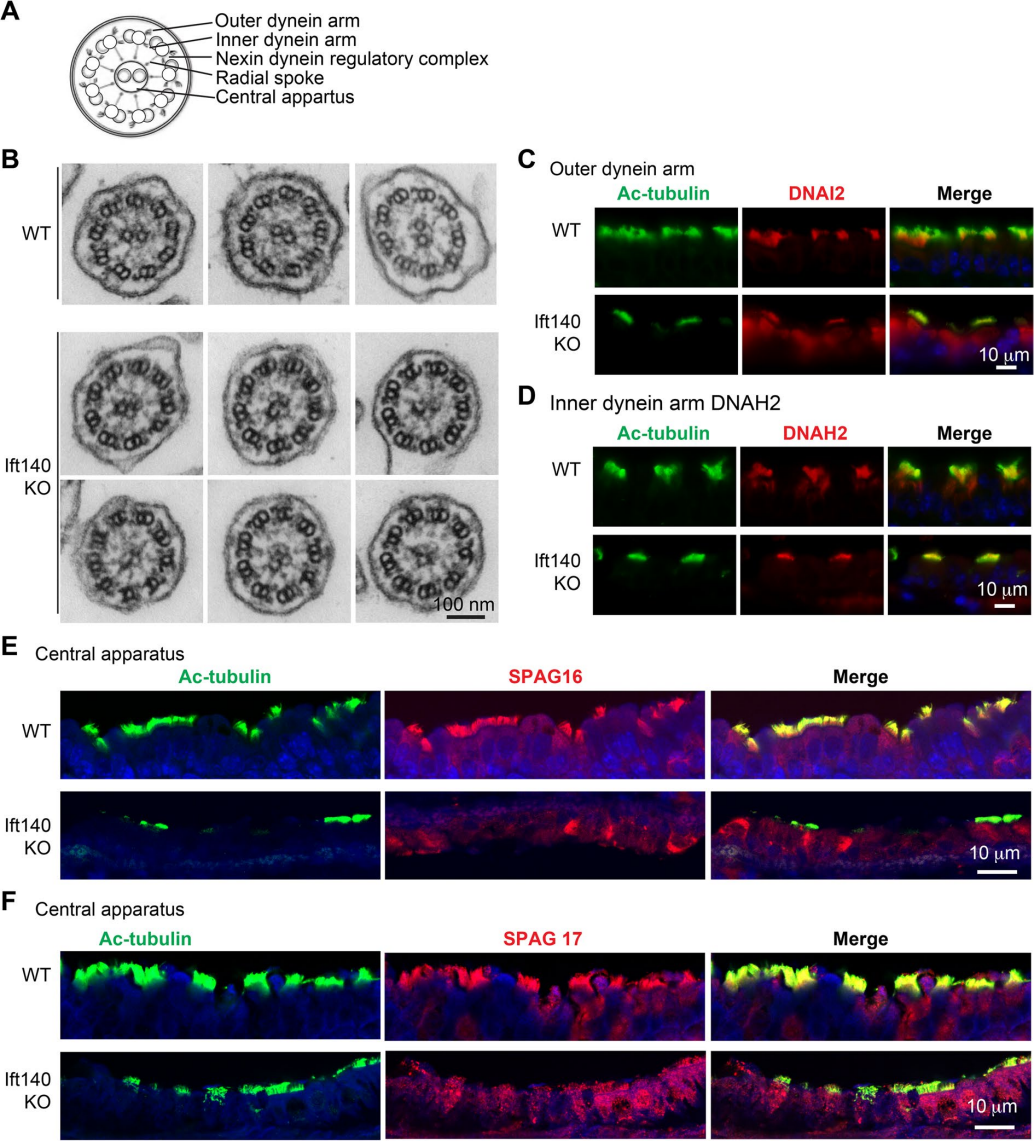
Inactivation of *Ift140* in motile cilia does not change the axonemal “9 + 2” structure, but affects the localization of the key central pair apparatus. **A** Cartoon showing normal representative motile cilia structure; **B** Cilia in *Ift140* cKO mTEC retain normal “9 + 2” axonemal structure as examined by TEM; **C** Outer dynein arm protein DNAI2 localization in TEC of control and *Ift140* cKO mice. In control mice, DNAI2 signal was present in the cilia. In the *Ift140* cKO mice, even though less cilia were present, DNAI2 signal remained in the cilia; **D** Inner dynein arm protein DNAH2 localization in TEC of control and *Ift140* cKO mice. In control mice, DNAH2 signal was present in the cilia. In the *Ift140* cKO mice, even though less cilia were present, DNAH2 also in the cilia; **E** Central pair protein SPAG16L localization in mTEC of control and *Ift140* cKO mice. In control mice, SPAG16L signal was present in the cilia. In the *Ift140* cKO mice, SPAG16L signal was largely present in the cytoplasm; **F** Central pair protein SPAG17 localization in mTEC of control and *Ift140* cKO mice. In control mice, SPAG17 signal was present in the cilia. In the *Ift140* cKO mice, SPAG17 signal was largely present in the cytoplasm

Motile cilia normally have a “9 + 2” axonemal structure (Fig. 8A). Although *Ift140* cKO tracheal cilia are shorter than controls and have decreased cilia beat frequency, ultrastructural examination of the axoneme revealed morphologically normal appearing inner and outer dynein arms. However, the central pair apparatus appeared abnormal. Ultrastructural examination showed variation in central apparatus configurations, ranging from normal, to the absence of one or both microtubules (Fig. 8B). To investigate the assembly of components within the axonemes, localization of DNAI2 and DNAH2, components of outer and inner dynein arm motors, respectively, and SPAG16L (Chlamydomonas PF20) and SPAG17 (Chlamydomonas PF6), key components of the central pair apparatus were assessed by immunofluorescent microscopy. DNAI2 and DNAH2 were present in both control and *Ift140* cKO mice cilia even though these cilia were shorter (Fig. 8C, D). In contrast, SPAG16L and SPAG17 were present in control cilia, but in mutant mice most of the SPAG16L and SPAG17 proteins appeared to be localized in the cytoplasm of the cells (Fig. 8E, F).

## Discussion

IFT plays a significant role in ciliogenesis in *Chlamydomonas* and other ciliated species, as well as in primary cilia formation in vertebrates [4, 47, 48]. The elimination of IFT A component IFT140 in *Chlamydomonas* prevents cilia assembly [49]. The present study highlights that IFT140 is essential for motile ciliogenesis in mammals. *Ift140* cKO mice that survived to adulthood were completely infertile, and produce fewer, slow swimming sperm cells with abnormal sperm heads and shorter flagella together with ultrastructural defects. A similar phenotype was observed in the motile cilia in the respiratory tract. *Ift140* cKO mTEC exhibited abnormal cilia formation, characterized by reduced cilia number, a shorter length and decreased ciliary beat frequency compared to controls. Interestingly, in the brain ventricles, motile cilia appeared to be less affected, possible due to a different assembly program for these cells, indicating a need for further investigation of IFT in ependymal cilia.

IFT140 is a member of the IFT-A complex that has previously been studied primarily in non-mammalian organisms [50, 51]. We previously showed that IFT140 was required for sperm motility [28]. In our current studies, we extended these studies to show that IFT140 is also required for motile cilia function in other cell types. In airway cilia, we observed abnormal particle accumulation and bulges along the ciliary shaft beneath the membrane, which are typical features of IFT-A mutations. We also found enlarged cilia tips, attributed to failure of retrograde transport. The observed reduced cilia length in the mutant cilia has been typically attributed to IFT-B mutants owing to decreased delivery of proteins during cilia assembly [52–55]. The mechanisms for inadequate cilia assembly leading to shorter cilia in the *Ift140* mutant is not known, but there are several potential interactions between components of IFT-A and IFT-B that may be disrupted [56].

The precise cargo handled by IFT140 is undefined. TEM of the short IFT140 mutant cilia suggests that the motor complexes for the inner and outer dynein arm do not require IFT140 (Fig. 8C, D). However, in the cilia cross sections the central apparatus ultrastructure appeared abnormal. Central apparatus protein SPAG16L and SPAG17 did not properly localize in the cilia (Fig. 8E, F). It is also uncertain if there are specific IFT for trafficking central apparatus components such as SPAG16L. Immunofluorescence localization identified these proteins in the cytoplasm, which could be due to a transport or docking defect.

In the present investigation, only the first generation of homozygous mutant mice survived to adulthood. These mice did not exhibit any gross abnormalities, such as hydrocephalus. However, all these homozygous mutant mice were completely infertile. Subsequently homozygous mutant mice in all generations analyzed in this study died before reaching three weeks of age, accompanied by a significant reduction in body weight. A potential contributing factor could be the acquisition of additional epigenetic mutations [57]. Motile cilia are also present in the fetal esophagus at around week 16 of gestation but disappear by birth [58]. It is less likely that the *Ift140* mutant mice died an abnormal system because all the mutant mice were normal until one week after birth.

The observed spermatogenesis defects in *Ift140*^*flox/flox*^*;FOXJ1*-Cre^±^ male mice are consistent with observations in a germ cell-specific *Ift140* knockout mice model [28]. This model revealed that IFT140 directly contributes to sperm flagella assembly, morphology and maintenance of normal sperm head morphology [28]. In addition to its direct impact on flagella assembly, another potential cause of aberrant spermatogenesis in the current *Ift140* cKO mice may stem from defects in the efferent duct. Mature spermatozoa produced in the seminiferous tubules pass through the efferent ducts, which reabsorb nearly 90% of the luminal fluids, during their transit to the epididymis. The efferent duct is lined with multiple motile ciliated cells, which are believed to play a crucial role in generating the luminal turbulence needed to prevent sperm aggregation [42–45, 59]. Disruption of motile cilia function in the efferent duct can lead to excessive fluid resorption, which leads to sperm aggregation and blockage of the ductal lumen. These ductal abnormalities cause fluid accumulation upstream and backflow into the seminiferous tubules, which results in seminiferous tubule dilation, epithelial degeneration and infertility. Although the inability to obtain adult *Ift140* cKO mice prevented further investigation, our findings demonstrate a significant reduction in cilia signals in the *Ift140* cKO mice. It is hypothesized that the multiciliated cells in the efferent ducts of the *Ift140* cKO mice are defective, contributing to blockage of the efferent ducts, enlargement of the seminiferous tubule lumens, and dramatic reduction in cauda epididymal sperm concentrations. To investigate the cause of female infertility, attempts were made to collect reproductive organs from *Ift140* cKO mice. Unfortunately, due to technical issues, the attempts were not successful.

The perinatal defects and motile ciliopathies may also share some features with human ciliopathies whose features have strong primary cilia defects but may also involve motility defects. IFT-A genetic mutations are causative of human ciliopathy syndromes including short-rib thoracic dysplasia, cranioectodermal dysplasia, asphyxiating thoracic dystrophy, nephronophthisis, Senior-Loken syndromes, and retinitis pigmentosa. The high mortality of patients with these syndromes has made it difficult to study motile cilia in these syndromes, but many of them have clinical features of motile ciliopathies, especially respiratory symptoms [18, 60–62], perhaps dependent on the specific gene variant. Many ciliopathies affecting IFT and primary cilia also have a high perinatal mortality [58]. Because the *Ift140* cKO mice exhibited growth retardation and premature death, it will be difficult to determine if causes of death are similar to that in complex human ciliopathies.

While male and female fertility and respiratory cilia were affected, the motile ependymal cilia may be unaffected, based on the absence of hydrocephalus. Hydrocephalus, characterized by the aberrant flow of cerebrospinal fluid, is another phenotype associated with motile cilia defects that is common in mice deficient in motile cilia genes but very rare in humans with PCD [40, 63]. The *Ift140* cKO mice did not exhibit any signs of gross hydrocephalus. Immunofluorescence staining also indicated that the cilia length in the brain ventricle epithelial cells appeared to be unaffected in the *Ift140* cKO (Supplemental Fig. 2). It is possible that IFT140 does not play a major role in motile ciliogenesis in brain ventricle epithelial cells.

The *Ift140* cKO also lacked evidence of developmental situs abnormalities. Left–right axis patterning has been established to be associated with *Foxj1* expression and the regulation of asymmetric genes in the embryonic nodal cells [30, 34, 64]. A recent study demonstrated that IFT140 was required for left–right patterning during early embryogenesis using a global Cre-driver [64]. One possibility is that the few affected mice in our model died at embryonic stage; another possibility is that our Cre line does not efficiently delete *Ift140* in the nodal cells. It is also possible that the number of animals analyzed in this study was relatively low and there were no mice with situs inversus by chance. The absence of left–right defects in the cKO mice may more likely be related to the role of IFT140 for central apparatus function, as suggested by loss of SPAG16L decreased central apparatus structures observed in sperm of our *Ift140* cKO mouse. The “9 + 0” motile cilia in nodal cells generate a leftward fluid flow through spinning, which serves as the primary signal for establishing left–right axis asymmetry [30, 34, 64–67]. In PCD and mouse models, gene mutations affecting the central pair do not result in left–right asymmetry defects [68].

Based on the observations in the present study, IFT140 is essential for motile ciliogenesis in selected organs with motile cilia. It is possible that short cilia with diminished cilia beat frequency are a feature of acquired ciliopathies, particularly attributed to cigarette smoke exposure, perhaps due to interruption of IFT proteins including IFT140 [69]. This study demonstrates that IFT not only plays a role in primary cilia formation but is also crucial for motile ciliogenesis in mammals. It also contributes in to identification the common and specific IFT molecular actors within the diverse cilia types. Even though we identified SPAG16L and SPAG17 to be potential cargos of IFT140 in assembling the motile cilia, due to technique limitations, we were not able to discover more targets in this study. With more sensitive proteomics approaches available, it becomes possible to dissect the IFT140 complex globally in these motile cilia. For example, Wallingford’s lab studied the IFT122 complex using the powerful DIFFRAC method [70]. The same method could be used to investigate the role of IFT140 and other IFT complexes in cilia assembly. Using affinity purification coupled with mass spectrometry, IFT140 interaction in HEK293 cells was also identified [71]. Normal IFT-A complex structure was also dissected by cryo-EM [51] as well as combined chemical cross-linking mass spectrometry and cryo-electron tomography with AlphaFold 2-based prediction [72]. Future studies of mutant IFT cilia using these methods will be instructive. In conclusion, our studies enriched the current knowledge in the IFT-A assembly and IFT train formation and can serve as a basis for future structural-function analyses.

## Materials and methods

### Generation of ***Ift140***^***flox/flox***^***; FOXJ1***-Cre mouse model

*Floxed Ift140* mice were generated by Dr. Gregory J. Pazour, University of Massachusetts Medical School [73]. Transgenic mouse lines, expressing improved Cre recombinase under the control of a 1.2 kilobase promoter region of the human Forkhead box J1 (*FOXJ1*) gene*,* were obtained from Dr. Michael J. Holtzman, Washington University School of Medicine in St. Louis [41]. Three to four-month-old *FOXJ1-Cre* males were crossed with three to four-month-old *Ift140* females to obtain *Ift140*^*flox*/+^*;* *FOXJ1-Cre*^±^ mice. Three to four-month-old *Ift140*^*flox*/+^*;* *FOXJ1-Cre*^±^ males were backcrossed with three- to four-month-old *Ift140*^*flox/flox*^ females again, and the *Ift140*^*flox/flox*^*;* *FOXJ1-Cre*^±^ were considered to be the homozygous knockout mice (cKO), and *Ift140*^*flox/*+^*;* *FOXJ1-Cre*^±^ mice were used as the controls. Mice were genotyped by PCR using multiplex PCR mix (Bioline, Cat No. BIO25043) and primers as indicated in the Supplemental Table 1. The presence of the *FOXJ1-Cre* allele was evaluated as described in Zhang et al. [41], and *Ift140* genotypes were determined as described previously [64].

### Tissue histology

Testis, epididymides, trachea, and other indicated tissues were fixed in 4% paraformaldehyde overnight. The tissues were embedded in paraffin, sectioned at 5 μm thickness, deparaffined, and stained with hematoxylin and eosin, using standard procedures. Slides were examined using a BX51 Olympus microscope (Olympus Corp., Melville, NY, Center Valley, PA), and photographs were taken with a ProgRes C14 camera (Jenoptik Laser, Germany).

### Mouse tracheal epithelial cell culture

Progenitor basal cells were isolated from the trachea from 3–4-week-old wild type, heterozygous, and *Ift140* cKO mice following incubation with pronase for 24 h, at 4 °C. Progenitor basal cells were expanded on collagen-coated supported membranes (Transwell, Corning, Cat. no. 3460) in custom made growth factor-containing media, with the additional use of dual SMAD inhibition over 5–7 days as described [37, 74]. Once confluent, the apical media was removed and cells were differentiated at air–liquid interface on Transwell membranes for up to four weeks prior to assay [75].

### Cilia beat frequency measurement

CBF was quantified as described by Horani et al., using the Sisson–Ammons Video Analysis (SAVA, Ammons Engineering) [37]. All cells that had visible and in-focus full length of cilia were considered for cilia length measurements. CBF was recorded in: (1) tracheas isolated from mice and the CBF was analyzed immediately; (2) tracheae isolated and shipped overnight in DMEM with 10% fetal calf serum to the Brody laboratory, and after transfer to mTEC media and placement tissue culture incubator; (3) cultured mTEC.

To quantify CBF from each type of cell preparations, cilia were imaged using a Nikon Ti inverted microscope enclosed in an environmental chamber held at 37 °C. Data were recorded and averaged using Sisson–Ammons Video Analysis software (SAVA, Ammons Engineering, Clio, MI) [37]. To record CBF of cells from tracheae, rings cut from trachea and cells scraped from the tracheal wall were placed on a glass microscope slide with mouse trachea culture media under a coverslip. Beat frequency was recorded from cell clusters containing ciliated cells using a 40X Hoffman modulation contrast lens (NAMC3, Nikon) or 20X phase contrast lens. CBF from each cluster was averaged and the data from each cluster was considered as a single data sample. CBF of mTEC cultures was quantified from recordings of five fields per Transwell insert using at 20X phase objective. The average CBF of each field was recorded as a single data point.

### Immunofluorescence staining

The indicated tissues from the control and cKO mice were fixed with 4% paraformaldehyde in 0.1 M phosphate buffered saline (PBS; pH 7.4), and 5 μm paraffin sections were made. Cultured mTEC were immunostained as previously described. The sections or cells were incubated with the indicated primary antibodies at 4 °C overnight. SPAG16L (1:300 dilution) and SPAG17 (1:300 dilution) antibodies were previously generated in our laboratory [76, 77]. Mouse acetylated alpha tubulin antibodies were purchased from Sigma-Aldrich (clone 6-11B-1, T6793. 1:300 dilution) and Proteintech (66,200–1-ig. 1:300 dilution). Slides were washed with PBS and incubated for 1 h at room temperature with Alexa 488-conjugated anti-mouse IgG secondary antibody (1:1000; Jackson ImmunoResearch Laboratories) or Cy3-conjugated anti-rabbit IgG secondary antibody (1:1000; Jackson ImmunoResearch Laboratories). Following secondary antibody incubation, the slides were washed three times with PBS, and mounted using VectaMount with DAPI (Vector Laboratories, Burlingame, USA), and sealed with a cover slip. Images were captured by confocal laser-scanning microscopy (Zeiss LSM 700). Some fluorescence images were captured using a spinning disc confocal system (Andor Dragonfly 200) mounted to an inverted microscope (Leica DMI8) with a 63X oil-immersion objective (Leica HC PL APO, NA 1.4) and a sCMOS camera (Andor Zyla). Consistent acquisition settings were applied across all color channels, and brightness/contrast adjustments were uniformly applied in ImageJ for display.

For detection of inner and outer dynein arm motor proteins in mouse lung, formalin-fixed, paraffin embedded tissues were sectioned and subjected to antigen retrieval by submersion in antigen retrieval buffer (Antigen unmasking solution, Vector, H-3300) and heating under pressure (Decloaking chamber, BioCare Medical) for 5 min, then cooled to room temperature. Tissue sections were incubated in blocking buffer (bovine serum albumin, 3%, in PBS) for 30 min at room temperature, then incubated in blocking buffer with the following primary antibodies at 4 °C overnight: Acetylated alpha-tubulin (1:5000, mouse clone 6-11B-1), DNAI2 (1:200, rabbit, Proteintech, Cat. no. 17533), DNAH2 (1:2000; Sigma, HPA067103). Slides were washed and tissues were incubated with secondary antibodies (Donkey anti-rabbit or Donkey anti-mouse with Alexa Fluor 488 or 555, Life Technologies) for 30 min at room temperature. Tissue sections were mounted in medium containing DAPI (Flouroshield, Sigma). Images were captured using a Leica 6000 microscope equipped with a cooled digital camera and Leica imaging software (LAS X).

### Electron microscopy

mTEC used for transmission electron microscopy analysis were cultured in Transwell filters and prepared as previously described by Horani et al. [37]. Briefly, cells were fixed with mix of 2% paraformaldehyde plus 2% glutaraldehyde in 100 mM sodium cacodylate buffer overnight at 4 °C. Cells were post fixed with osmium tetroxide (1%, Ted Pella, 18,459), stained in uranyl acetate (1%, Electron Microscopy Sciences, 22,400–1), embedded in Eponate 12 resin (Ted Pella, 18,005). Sections (95 nm) were imaged on grids using a JOEL 1200 EX TEM.

### Cilia length measurement

Cilia length was measured in fixed mTEC cultures that were immunostained and parallel samples prepared for transmission electron microscopy. In all samples, cilia length was measured by image J (FIJI) using the distant measurement tool. Cells cultured on Transwell membranes were fixed and immunostained for acetylated alpha-tubulin, scraped from membranes and mounted under coverslips. All cells that had visible and the full length of cilia in focus were considered for cilia length measurements. A total of 40–50 cilia in multiple fields were measured for control (WT and *Ift140*^+*/flox*^*;* *FOXJ1-Cre*^±^) and *Ift140* deficient (*Ift140*^*flox/flox*^*;* *FOXJ1-Cre*^±^) genotypes. Cilia length was measured from 17 to 19 different electron micrographs of the same genotype.

### Statistical analysis

Graphs were created using Microsoft Excel and GraphPad Prism. Statistical analyses were performed using Student’s *t* test. P > 0.05 was considered as not significant and by convention *p < 0.05, **P < 0.01. Multiple groups were compared using the Kruskal–Wallis test and Dunn’s multiple comparison test.

## Supplementary Information

Below is the link to the electronic supplementary material.

Supplemental Figure 1. Generation of Ift140 flox/flox; FOXJ1-Cre mouse models. A. Breeding strategy for the generation of mice with targeted Ift140 in motile cilia. A FOXJ1-Cre line was used to cross to the floxed Ift140 mice. B. Representative genotyping results by PCR showing the wild-type allele, floxed Ift140 allele and FOXJ1-Cre allele. PF: PCR forward primer; PR: PCR reverse primer. Two loxP sites were added to the genome flanking exon 7. The PCR product from the floxed allele was larger than from the wild-type allele. Supplemental Figure 2. Normal ciliogenesis in the brain ventricle ependymal cells of the Ift140 cKO mice. Immunofluorescence staining was conducted in brain sections from control and Ift140 cKO mice using an anti-acetylated tubulin antibody. Similar signal was observed in cilia of both control and Ift140 cKO mice. Supplemental Figure 3. Normal kidney and liver histology in the control and Ift140 cKO mice. A Histology of the kidney from the control and Ift140 cKO mice. B Histology of liver from the control and Ift140 cKO mice. Supplemental Figure 4. Analysis of the cilia formation in the cultured mouse tracheal epithelial cells. Tracheal epithelial cells from control and Ift140 cKO mice were cultured and the cells were stained with anti-α–tubulin and anti-centrin antibodies. Notice that only scatterd signal was detected in Ift140 cKO mice. Supplemental Figure 5. Reduced cilia beating frequency in freshly isolated trachea of the Ift140 cKO mice. Tracheas were isolated from the control and Ift140 cKO mice and CBF (Hz) was immediatelly analyzed. Control: N=6 trachea, 202 regions were measured from tissue cut from each trachea, and 6-33 regions of interest (ROl) per trachea were analyzed. cKO: N=3 trachea, 109 regions were measured from tissue cut from each trachea, and 6-30 regions of interest (ROl) per trachea were analyzed. Supplemental Figure 6. Additional TEM images showing abnormal protein trafficking in the cultured tracheal epithelial cells (mTEC) from Ift140 cKO. The arrows point to the particles abnormally accumulated in the cilia. Supplemental Table 1: Primers for genotyping the mice. Supplementary file1 (PPTX 41567 KB)

Supplemental movie 1. Representative movies of sperm from control mice. Supplemental movie 2. Representative movies of sperm from Ift140 cKO mice. Supplemental movie 3. Representative movies of ciliary beating in tracheal epithelial cell cultures from a control mouse. Supplemental movie 4. Representative movies of ciliary beating in tracheal epithelial cell cultures from an Ift140 cKO mouse. Supplementary file2 (PDF 100056 KB)

## Data Availability

The data is available from the corresponding author upon reasonable request.
