# Supplementary material for: Role of intraflagellar transport protein IFT140 in the formation and function of motile cilia in mammals
Source: Cell Mol Life Sci. 2025 May 10;82(1):198. doi: 10.1007/s00018-025-05710-z (PMC12065702; doi:10.1007/s00018-025-05710-z)
Supplement: Supplementary file 1 — Supplemental Figure 1. Generation of Ift140 flox/flox; FOXJ1-Cre mouse models. A. Breeding strategy for the generation of mice with targeted Ift140 in motile cilia. A FOXJ1-Cre line was used to cross to the floxed Ift140 mice. B. Representative genotyping results by PCR showing the wild-type allele, floxed Ift140 allele and FOXJ1-Cre allele. PF: PCR forward primer; PR: PCR reverse primer. Two loxP sites were added to the genome flanking exon 7. The PCR product from the floxed allele was larger than from the wild-type allele. Supplemental Figure 2. Normal ciliogenesis in the brain ventricle ependymal cells of the Ift140 cKO mice. Immunofluorescence staining was conducted in brain sections from control and Ift140 cKO mice using an anti-acetylated tubulin antibody. Similar signal was observed in cilia of both control and Ift140 cKO mice. Supplemental Figure 3. Normal kidney and liver histology in the control and Ift140 cKO mice. A Histology of the kidney from the control and Ift140 cKO mice. B Histology of liver from the control and Ift140 cKO mice. Supplemental Figure 4. Analysis of the cilia formation in the cultured mouse tracheal epithelial cells. Tracheal epithelial cells from control and Ift140 cKO mice were cultured and the cells were stained with anti-α–tubulin and anti-centrin antibodies. Notice that only scatterd signal was detected in Ift140 cKO mice. Supplemental Figure 5. Reduced cilia beating frequency in freshly isolated trachea of the Ift140 cKO mice. Tracheas were isolated from the control and Ift140 cKO mice and CBF (Hz) was immediatelly analyzed. Control: N=6 trachea, 202 regions were measured from tissue cut from each trachea, and 6-33 regions of interest (ROl) per trachea were analyzed. cKO: N=3 trachea, 109 regions were measured from tissue cut from each trachea, and 6-30 regions of interest (ROl) per trachea were analyzed. Supplemental Figure 6. Additional TEM images showing abnormal protein trafficking in the cultured tracheal epithelial cells ( [file 18_2025_5710_MOESM1_ESM.pptx]

## Slide 1
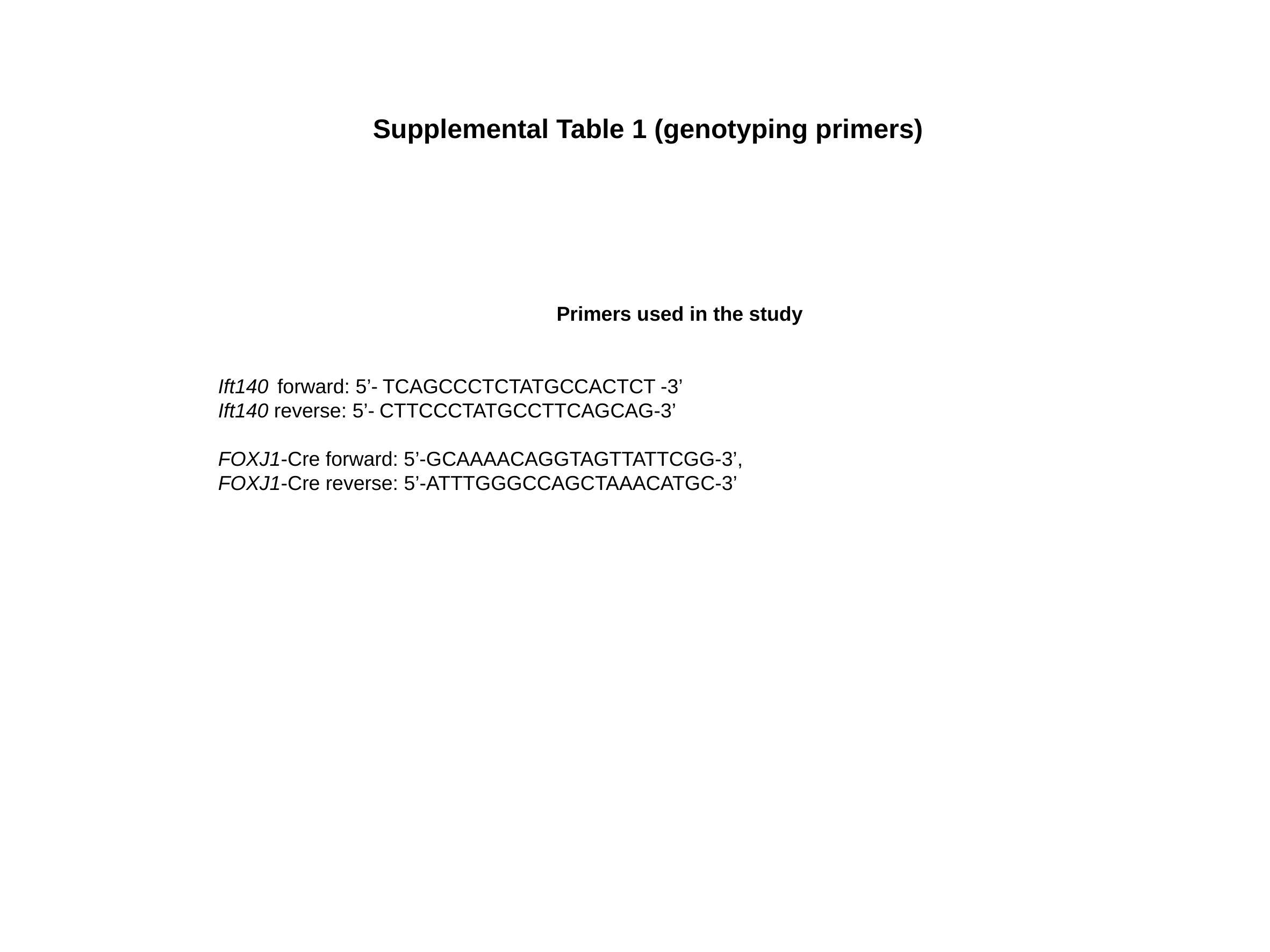

# Supplemental Table 1 (genotyping primers)
Primers used in the study
Ift140 forward: 5’- TCAGCCCTCTATGCCACTCT -3’
Ift140 reverse: 5’- CTTCCCTATGCCTTCAGCAG-3’
FOXJ1-Cre forward: 5’-GCAAAACAGGTAGTTATTCGG-3’,
FOXJ1-Cre reverse: 5’-ATTTGGGCCAGCTAAACATGC-3’

## Slide 2
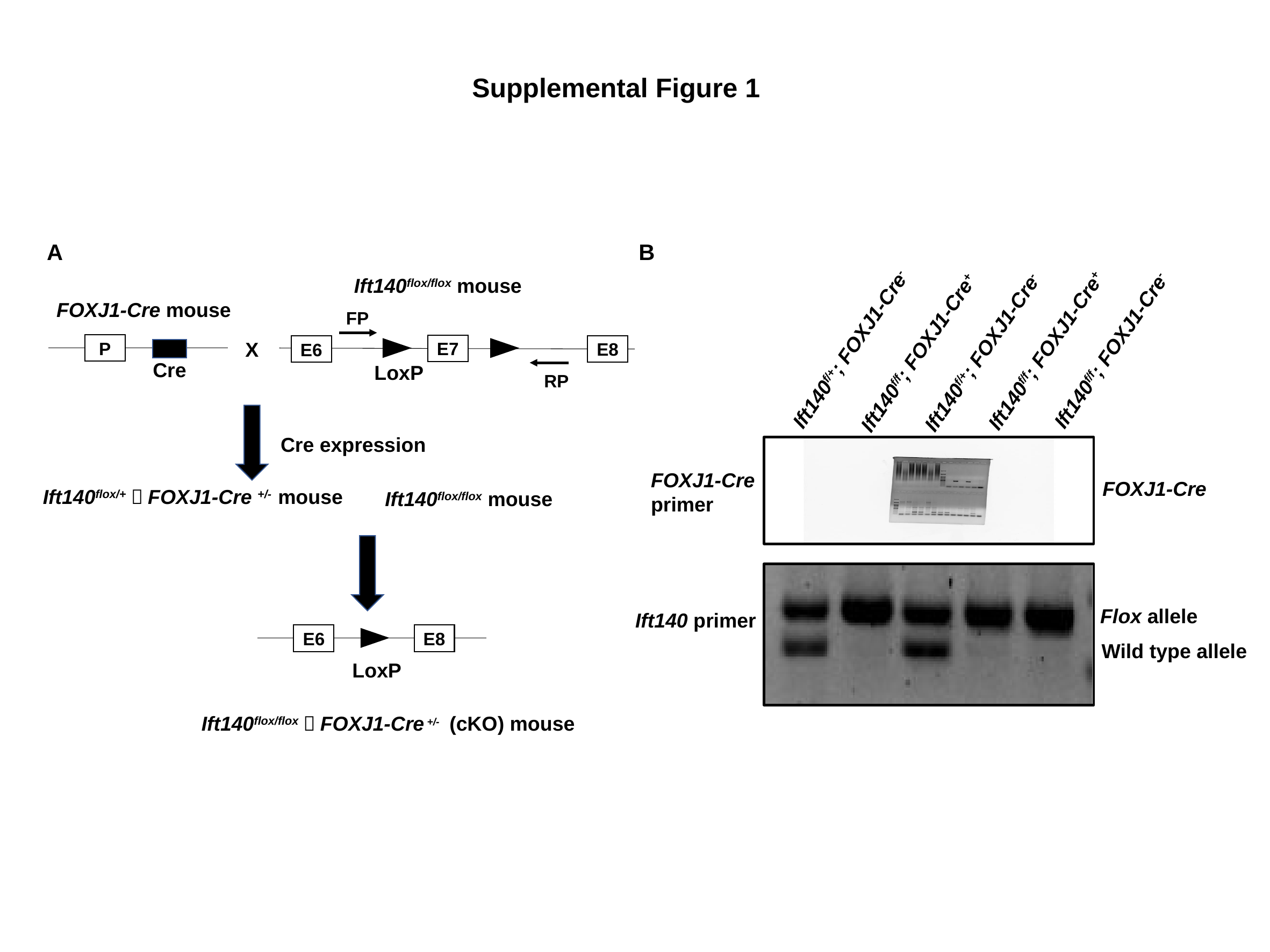

# Supplemental Figure 1
A
B
Ift140flox/flox mouse
FOXJ1-Cre mouse
FP
Ift140f/+; FOXJ1-Cre-
Ift140f/f; FOXJ1-Cre-
Ift140f/f; FOXJ1-Cre+
Ift140f/+; FOXJ1-Cre-
Ift140f/f; FOXJ1-Cre+
X
P
E7
E8
E6
Cre
LoxP
LoxP
RP
Cre expression
FOXJ1-Cre
primer
FOXJ1-Cre
Ift140flox/+；FOXJ1-Cre +/- mouse
Ift140flox/flox mouse
Flox allele
Ift140 primer
E8
E6
Wild type allele
LoxP
Ift140flox/flox；FOXJ1-Cre +/- (cKO) mouse

## Slide 3
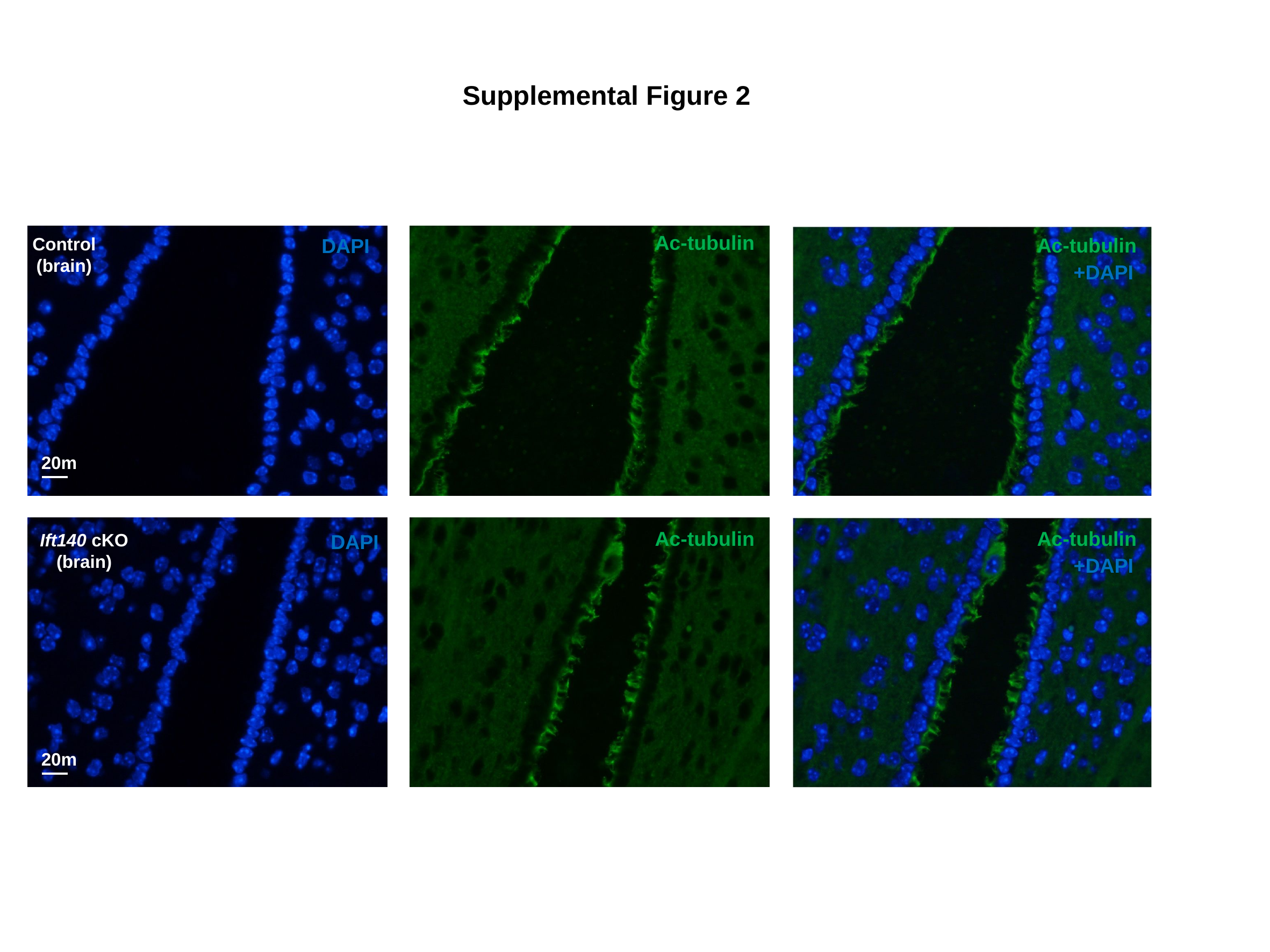

# Supplemental Figure 2
Ac-tubulin
Control (brain)
Ac-tubulin
DAPI
+DAPI
Ac-tubulin
Ac-tubulin
Ift140 cKO (brain)
DAPI
+DAPI

## Slide 4
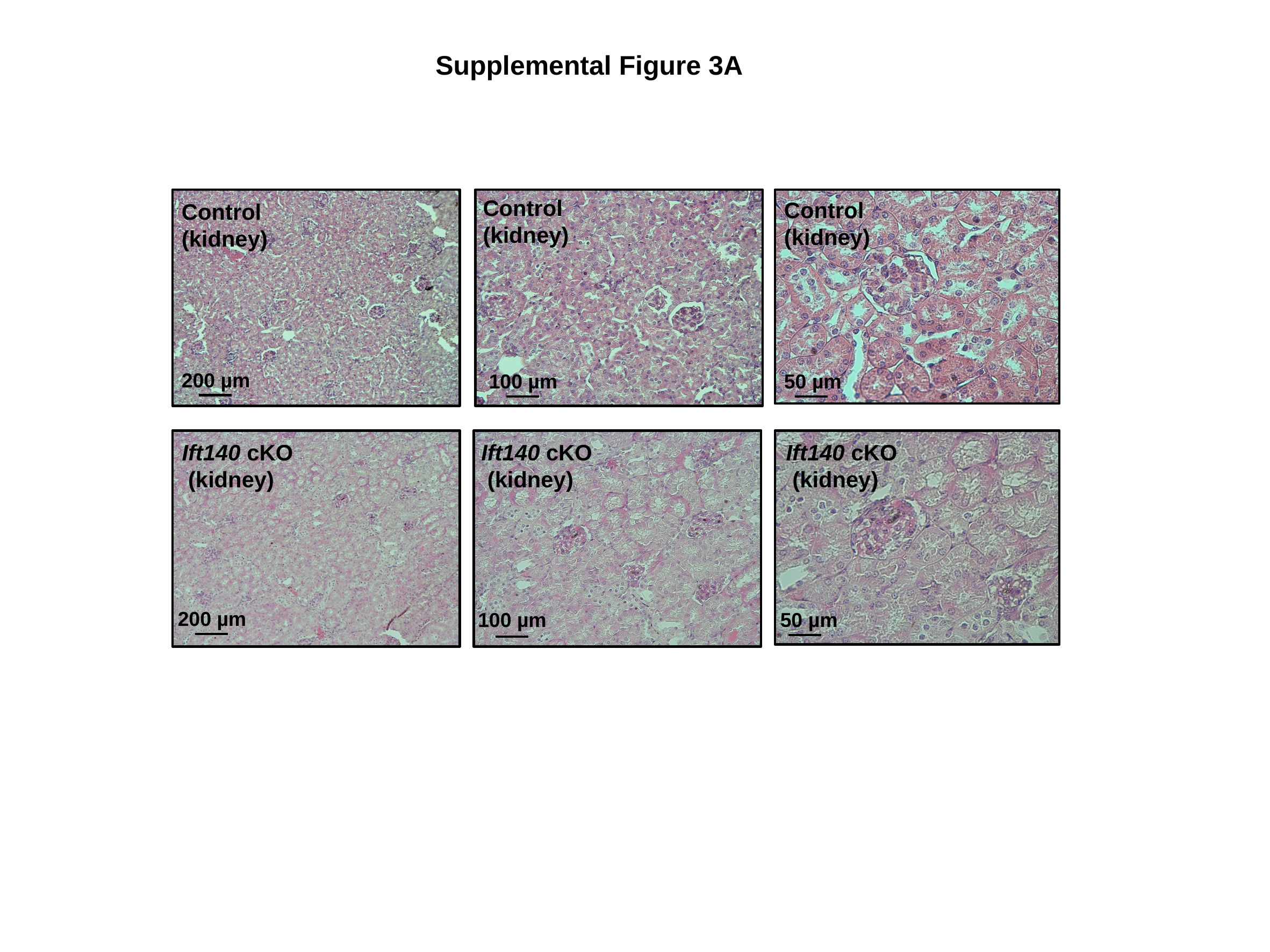

# Supplemental Figure 3A
Control
(kidney)
Control
(kidney)
Control
(kidney)
200 µm
50 µm
100 µm
Ift140 cKO
 (kidney)
Ift140 cKO
 (kidney)
Ift140 cKO
 (kidney)
200 µm
50 µm
100 µm

## Slide 5
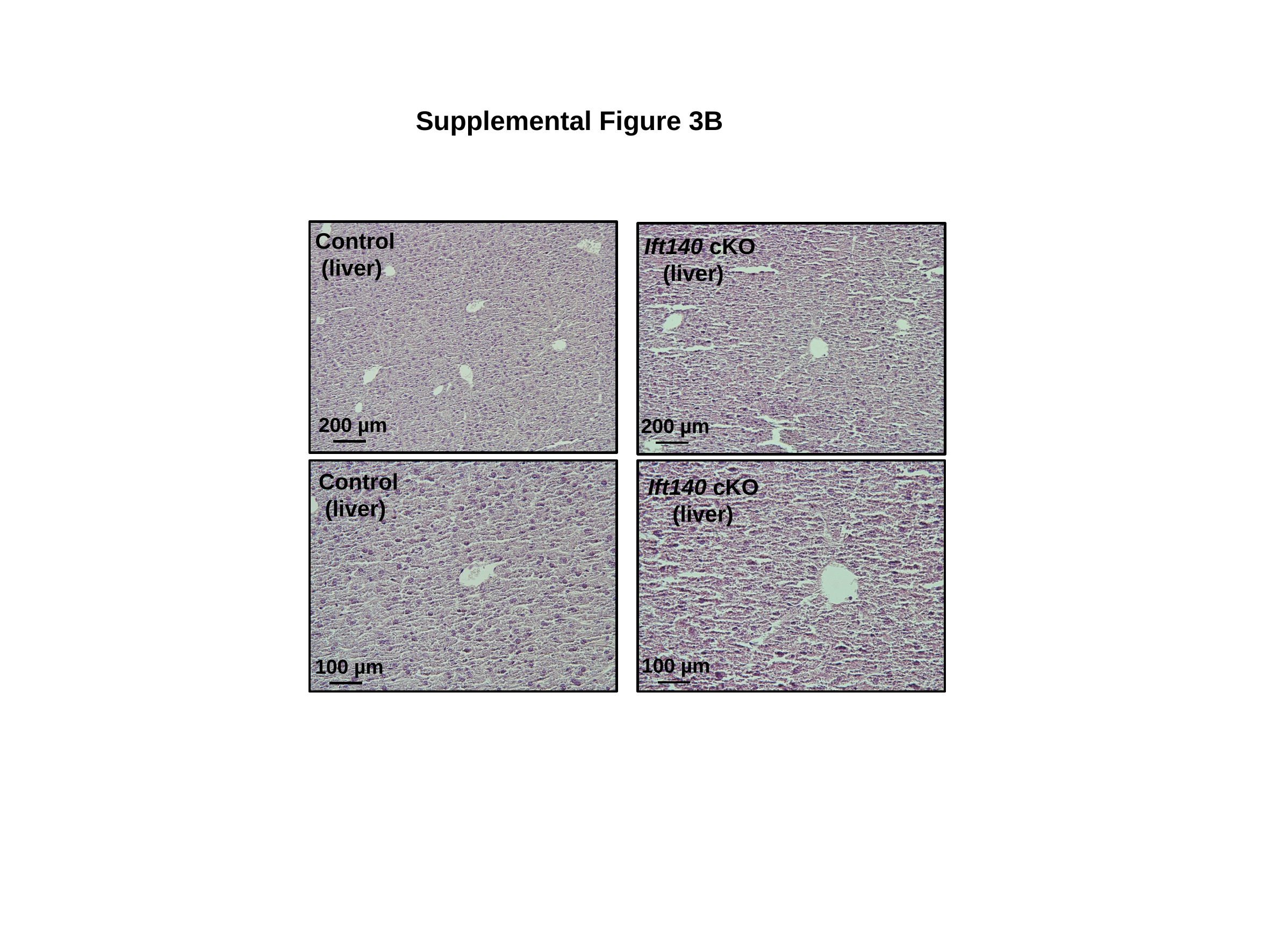

# Supplemental Figure 3B
Control
 (liver)
Ift140 cKO
 (liver)
200 µm
200 µm
Control
 (liver)
Ift140 cKO
 (liver)
100 µm
100 µm

## Slide 6
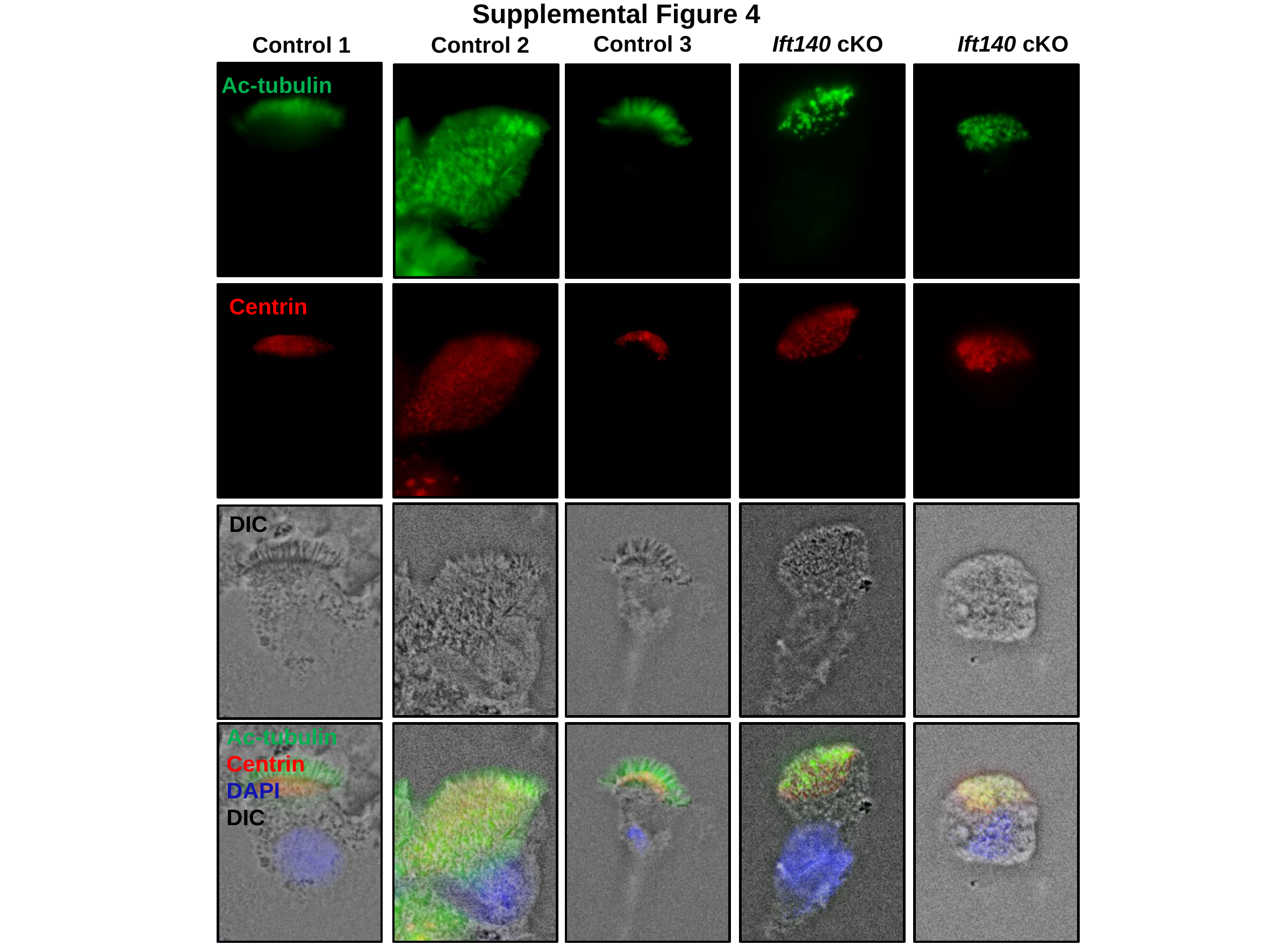

Supplemental Figure 4
Control 3 Ift140 cKO Ift140 cKO
Control 1 Control 2
Ac-tubulin
Centrin
DIC
Ac-tubulin
Centrin
DAPI
DIC

## Slide 7
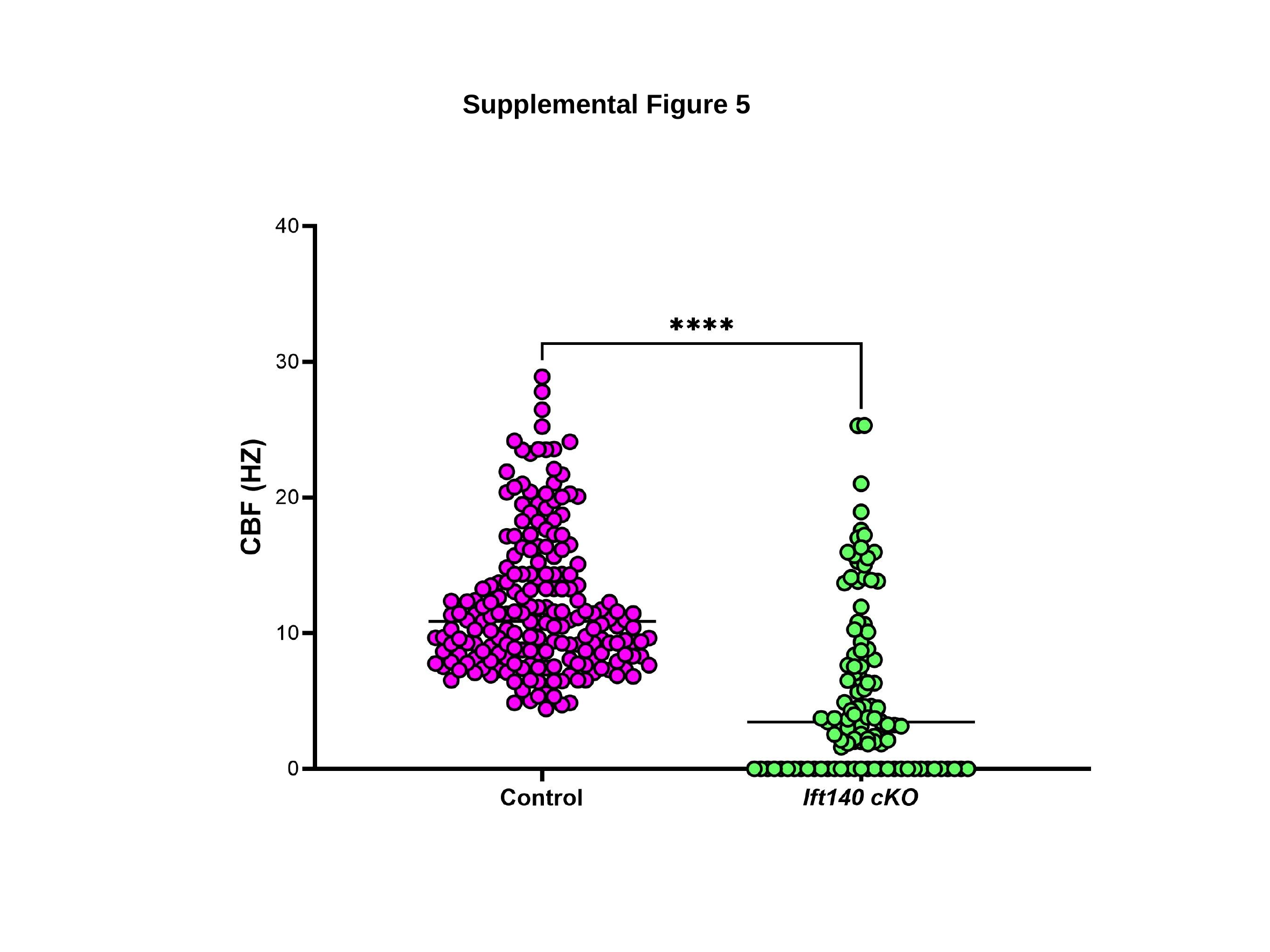

Supplemental Figure 5

## Slide 8
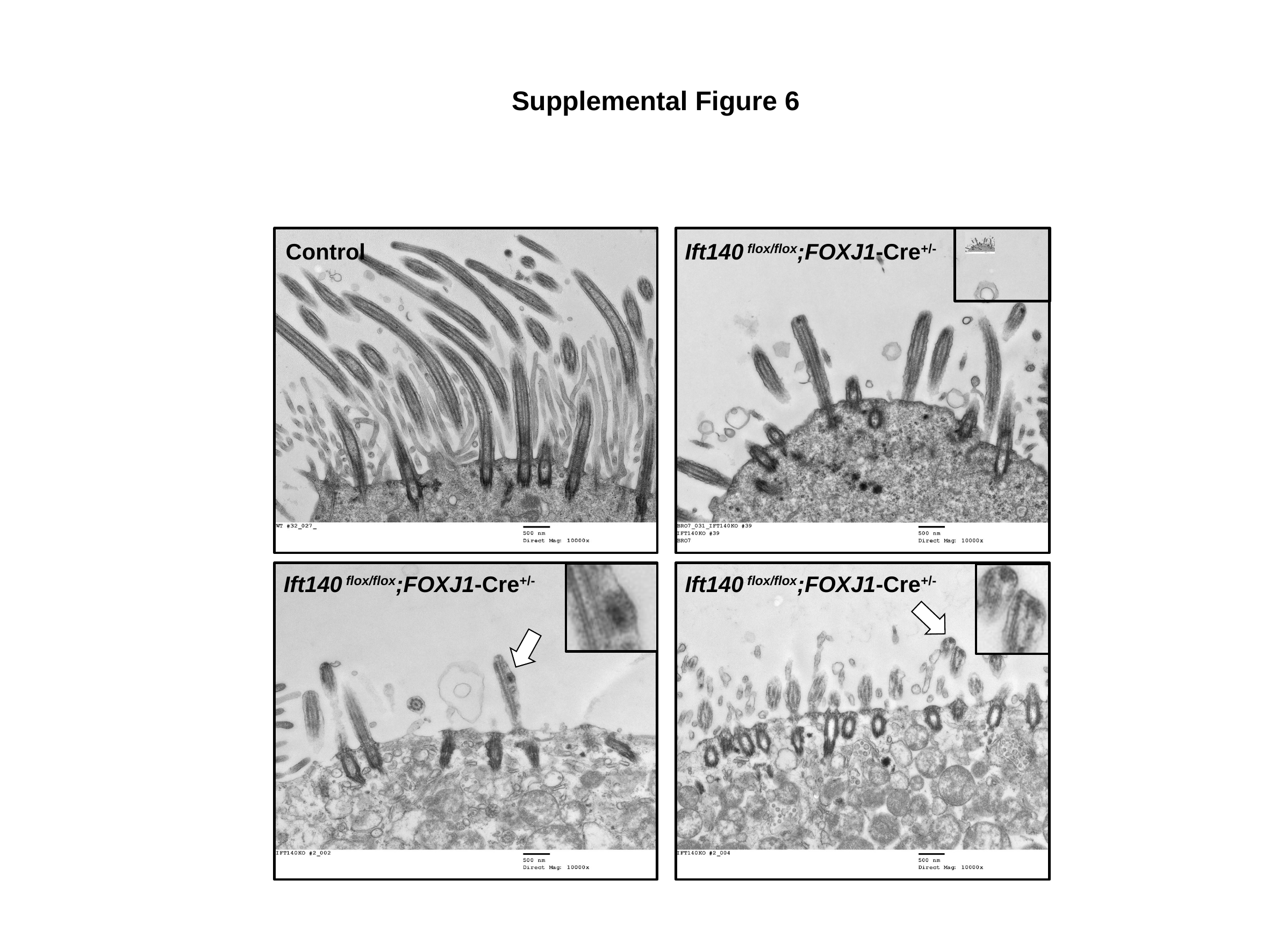

Supplemental Figure 6
Ift140 flox/flox;FOXJ1-Cre+/-
Control
Ift140 flox/flox;FOXJ1-Cre+/-
Ift140 flox/flox;FOXJ1-Cre+/-
