# Supplementary material for: Role of intraflagellar transport protein IFT140 in the formation and function of motile cilia in mammals
Source: Cell Mol Life Sci. 2025 May 10;82(1):198. doi: 10.1007/s00018-025-05710-z (PMC12065702; doi:10.1007/s00018-025-05710-z)
Supplement: Supplementary file 2 — Supplemental movie 1. Representative movies of sperm from control mice. Supplemental movie 2. Representative movies of sperm from Ift140 cKO mice. Supplemental movie 3. Representative movies of ciliary beating in tracheal epithelial cell cultures from a control mouse. Supplemental movie 4. Representative movies of ciliary beating in tracheal epithelial cell cultures from an Ift140 cKO mouse. Supplementary file2 (PDF 100056 KB) [file 18_2025_5710_MOESM2_ESM.pdf]

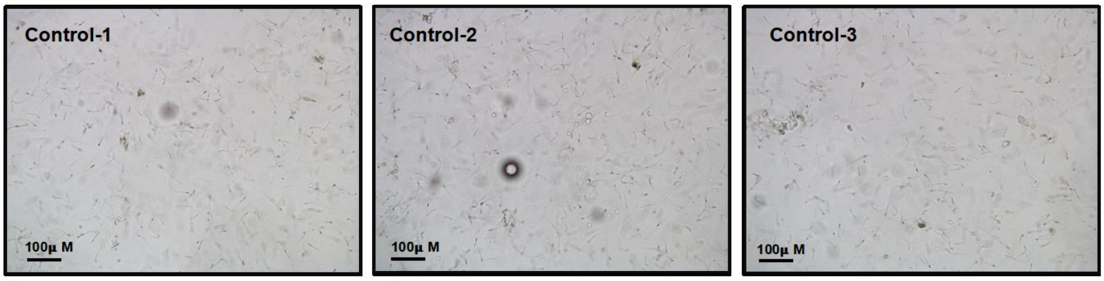

Supplemental movie 1. Representative movies of sperm from control mice.

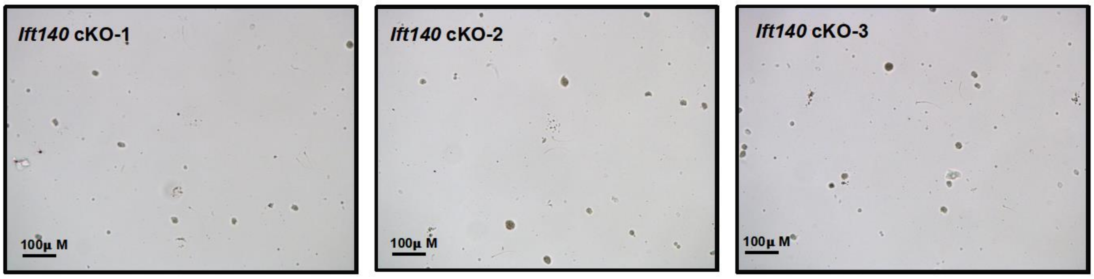

Supplemental movie 2. Representative movies of sperm from *Ift140* cKO mice.

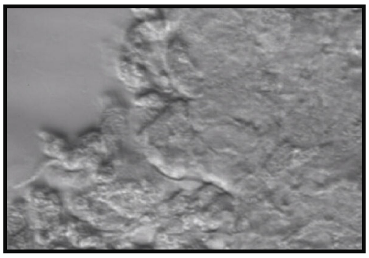

Supplemental movie 3. Representative movies of ciliary beating in tracheal epithelial cell cultures from a control mouse.

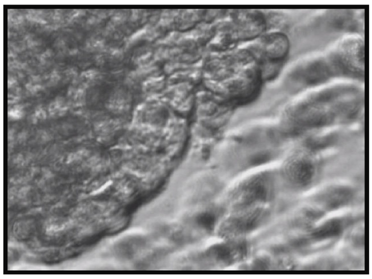

Supplemental movie 4. Representative movies of ciliary beating in tracheal epithelial cell cultures from an *Ift140* cKO mouse.

Ift140 forward: 5’- TCAGCCCTCTATGCCACTCT -3’
Ift140 reverse: 5’- CTTCCCTATGCCTTCAGCAG-3’

FOXJ1-Cre forward: 5’-GCAAAACAGGTAGTTATTCGG-3’,
FOXJ1-Cre reverse: 5’-ATTTGGGCCAGCTAAACATGC-3’

Supplemental Table 1: Primers for genotyping the mice.
